# Supplementary material for: Label-free determination of diffusion coefficients at the nanoscale through modelling of the Surface Plasmon Resonance signal
Source: PLoS One. 2025 Jan 7;20(1):e0312594. doi: 10.1371/journal.pone.0312594 (PMC11706461; doi:10.1371/journal.pone.0312594)
Supplement: S1 File — (PDF) [file pone.0312594.s001.pdf]

Oct 17, 2024

Label-Free determination of diffusion coefficients at the nanoscale through modelling of the Surface Plasmon Resonance signal S1_file

DOI
dx.doi.org/10.17504/protocols.io.x54v927rml3e/v1

Gabriele Antonio Zingale[1], Irene Pandino[1], Damiano Calcagno[1], Maria Luisa Perina[2], Nunzio Tuccitto[2], Giuseppe Grasso[2]

[1]IRCCS Fondazione Bietti, Rome, Italy;
[2]Department of Chemical Sciences, University of Catania, Viale Andrea Doria 6, 95125, Catania, Italy

Gabriele Antonio Zingale: ¶ These authors contributed equally to this work.;
Irene Pandino: ¶ These authors contributed equally to this work.
Giuseppe Grasso: Corresponding author

Gabriele Antonio Zingale
IRCCS Fondazione Bietti, Rome

OPEN ACCESS

DOI: **dx.doi.org/10.17504/protocols.io.x54v927rml3e/v1**

Protocol Citation: Gabriele Antonio Zingale, Irene Pandino, Damiano Calcagno, Maria Luisa Perina, Nunzio Tuccitto, Giuseppe Grasso 2024. Label-Free determination of diffusion coefficients at the nanoscale through modelling of the Surface Plasmon Resonance signal S1_file. **protocols.io https://dx.doi.org/10.17504/protocols.io.x54v927rml3e/v1**

Manuscript citation:
Customer code: PLOS2022
This protocol is associated with a PLOS ONE Lab Protocol Submission (PONE-D-24-24894R1 Label-Free determination of diffusion coefficients at the nanoscale through modelling of the Surface Plasmon Resonance signal)

License: This is an open access protocol distributed under the terms of the **Creative Commons Attribution License**, which permits unrestricted use, distribution, and reproduction in any medium, provided the original author and source are credited

Protocol status: Working
We use this protocol and it's working

Created: June 20, 2024

Last Modified: October 17, 2024

Protocol Integer ID: 102146

Keywords: SPR, mass transport, Taylor diffusion analysis, Python script, microfluidic channels

Funders Acknowledgement:
MIUR, PRIN
Grant ID: P2022AW2H9
Pharma-HUB - HUB per il riposizionamento di farmaci nelle malattie rare del sistema nervoso in età pediatrica”
Grant ID: T4-AN-04
LazioInnova
Grant ID: A0375-2020-36591

Abstract

Surface plasmon resonance (SPR) is normally used to measure the kinetic parameters of biomolecular interactions between a molecule immobilized on a gold surface and another one flowing in a microfluidic channel above the surface. During the SPR measurements, convection-diffusion phenomena occur inside the microfluidic channels, but they are generally minimized by appropriate experimental setup in order to obtain diffusion free kinetic parameters of the molecular interactions. In this work, for the first time, a commercial SPR apparatus has been used to obtain non canonical scientific parameters. Indeed, a specifically designed SPR experimental setup is described for carrying out measurements of the diffusion coefficient (*D*) of molecules in solutions. The high precision and reproducibility of the approach, as well as the wide applicability of the newly proposed SPR based method for the measurement of *D* of many different molecules and biomolecules, are here demonstrated and illustrated in detail.

Title

1 Label-Free determination of diffusion coefficients at the nanoscale through modelling of the Surface Plasmon Resonance signal

Materials and Methods

2 **1. Instrumental setting and features**

The diffusion experiments can be performed with any kind of SPR system.[1] This method was created and perfected using a multiparametric Bionavis SPR Navi 210A instrument equipped with two separate and parallel microfluidic channels, either used as a reference channel or as a sample channel.

The system mounts two distinct lasers at two different wavelengths, 670 nm and 785 nm respectively. Both wavelengths can be used for the purpose, indeed no substantial differences were seen in the evolution of the SPR signal during the time course of the experiments. The gold sensor-chips herein used are made up by 50 nm of gold and 2 nm of chromium deposited on a glass substrate. They are 20 mm long, 12 mm wide and 0.55 mm thick, whereas the area in contact with the flow is of 12 mm^2. The whole SPR Navi 210A fluidic system comprises a 1 μL flow cell with its tubing connectors, a dual-channel syringe pump (buffer pump) operating two independent flow-paths, one for each of the two measurement chambers (Fig. S1). Liquid flows from the buffer container through the injection valve system to the measurement chambers in the flow cell and out to the waste bottle. A single syringe pump is used for automatic loading and cleaning of sample loops. The PEEK tubes connecting the
loop to the flow cell have all an internal diameter of 254 μm.

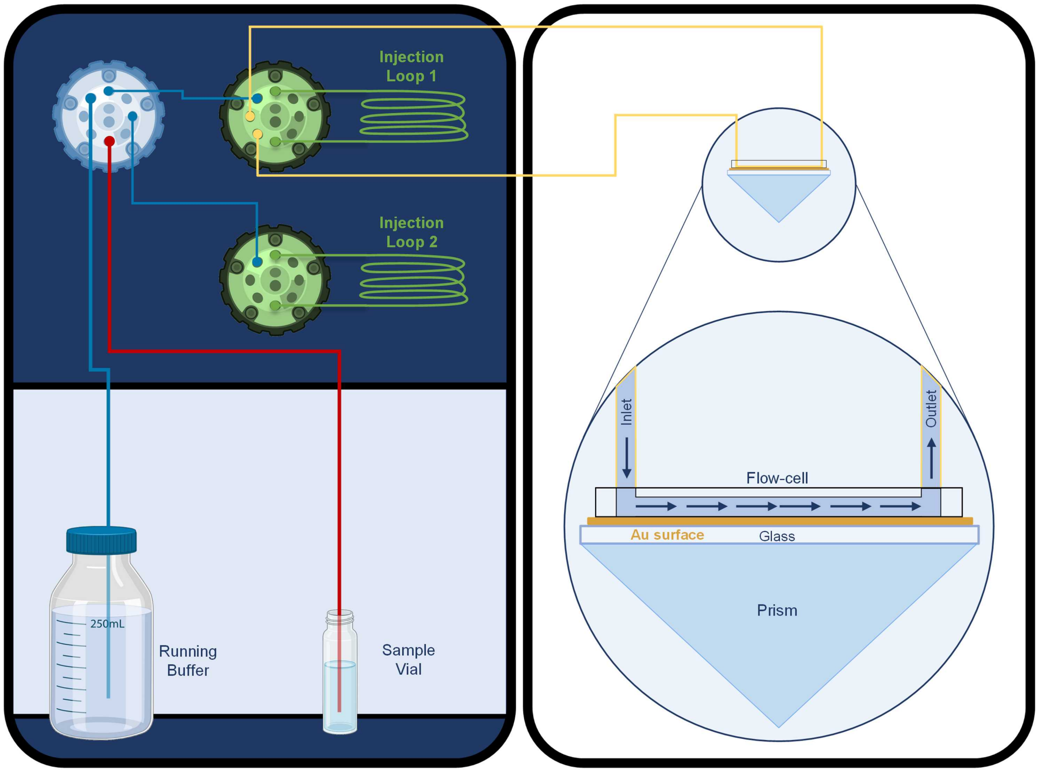

Fig. S1 Schematic representation of an SPR system.The flow within the microfluidic channels is controlled by a series of pumps and valves which direct the flow towards the flow cell or waste. An independent pump usually controls the injection profile through the loop.

The feasibility of the method relies on the size of the microfluidic tubing used. Longer and narrower tubing are preferred but they are limited by the instrument's pump features to operate a constant pressure, and therefore a flow rate, within the entire system. Moreover, the longer the tubing the longer the time needed to carry out an experiment. Therefore, clearly assuming to work in laminar regime conditions, the choice of the dimensions and length of the PEEK tubing is dictated by the compromise between instrumental characteristics and the time available to perform the experiments.

In the method herein described, a setting that allows to modulate the length of the PEEK tubing by connecting in series different units of 35 cm each was used. Since the purpose is to determine the diffusion coefficient (D) of molecules of different sizes, the length of the channel within which the analyte flows is of fundamental importance. In particular, small molecules (e.g. heavy water, ethanol, glycine) diffuse rapidly, therefore a single 35 cm 254 μm i.d. channel and a flow rate of 20 μL/min is enough to guarantee the diffusion of these molecules. On the other hand, larger molecules have a lower diffusion and, at the same tubing i.d. and flow rate, longer tubes (i.e. connecting two 35 cm tubes in series) are needed for the phenomenon to properly occur. Nominal flow rates of the injected solution can be set from 0 to 6000 μL/min.

3 **2. Running buffer preparation**

The running buffer is the solution used as a carrier throughout the experiment. The versatility of the method and the instrumentation allow to use any kind of solution as running buffer (e.g. MilliQ water, Phosphate Buffered Saline (PBS), tris(hydroxymethyl)aminomethane (TRIS)) with the possibility of modulating other parameters such as pH, ionic strength, temperature etc. It is crucial that all the buffers and solutions used are freshly prepared, filtered using a sterile syringe filter (0.22 μm) and degassed for at least 15 minutes in an ultrasonic bath before the injection into the SPR system to avoid the presence of dust and/or air bubbles that could affect the SPR response signal (Fig. S2). It is also advisable to use clean glass containers for buffer preparation and storage.

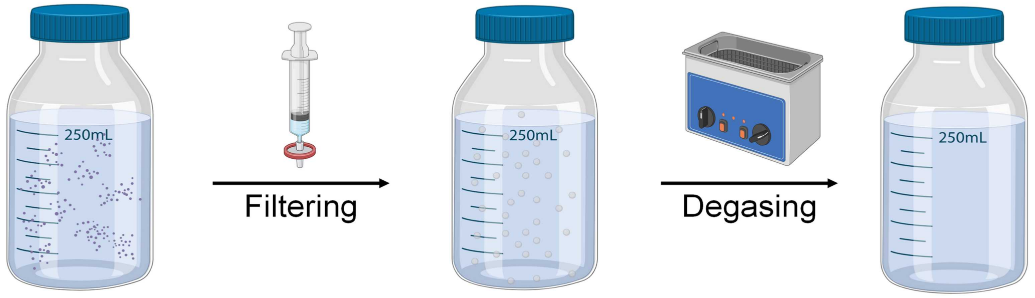

Fig. S2 Scheme of the running buffer preparation. The buffer is first filtered using a syringe and a 0.22 μm filter and then degassed with an ultrasonic bath for at least 15 minutes to remove any air bubbles present in the solution.

4

3. Conditioning of the microfluidic system

Conditioning of the microfluidic system is a fundamental part of the experimental procedure. It is made as a very first injection and performed for each analyte of interest before starting the related diffusion experiments. This procedure is essential for two reasons: first, it is necessary to condition the microfluidic tubes, including the loops, used for the sample injection; second, it is necessary to verify that the interactions of the analyte with the gold chip surface are negligible. Indeed, in an ideal diffusion experiment, after the passage of the analyte into the flow cell, the SPR signal must return to the baseline. In fact, it is important to highlight that for a non-interacting analyte, the SPR curve should have a rectangular-like shape just due to the change in the refractive index of the solution containing the analyte with respect to the pure running buffer. However, even for a non-interacting analyte injected with a rectangular pulse (Fig. S3, left panel), the shape of the curve is not strictly rectangular, but sigmoidal (Fig. S3, right panel). This is confirmed for all non-interacting analytes and it is due to the analyte diffusion occurring inside the tubing system.

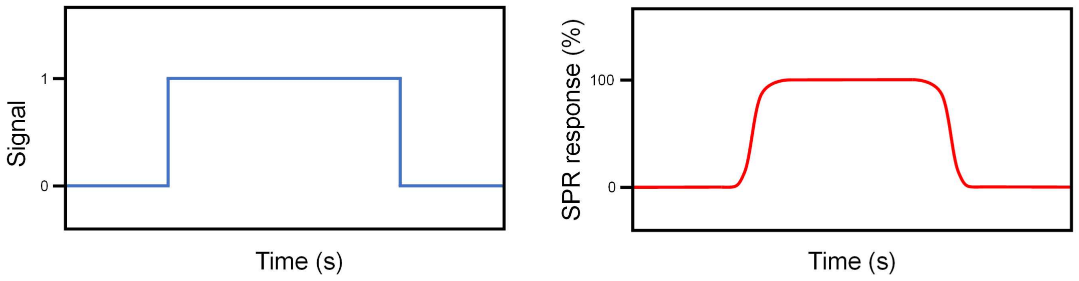

Fig. S3 Comparison between the injection pulse (left, blue line) and the SPR signal (right, red line) obtained as a function of time.

5 **4. Functionalization of the gold sensor-chip**

The nature of the molecule of interest defines the type of surface on which experiments will be performed, it can be a naked gold sensor-chip surface (Fig. S4, left) or a functionalized one (Fig. S4, right).

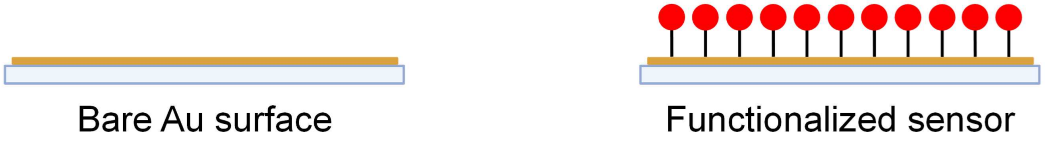

Fig. S4 General and schematic comparison between a bare gold sensor (left) and a functionalized one (right).

The former is particularly suitable for D measurements of relatively small and inert molecules as they do not interact with the gold sensor surface. Some features of the molecules that fall into this class are: molecular weight lower than 500 Da, absence of thiol groups and absence of a net positive charge. This is the case for inorganic ions, dissolved gases, small organic molecules (e.g., drugs, alcohols etc.) and small biomolecules (e.g., most amino-acids, small peptides, neurotransmitters, lipids etc.).

On the contrary, functionalized gold surfaces are needed for D measurements of larger molecules and, in particular, for peptides and proteins. This is because the interaction of these species with the gold sensor surface would be a limit of the SPR method here described. Basically, proteins give electrostatic, hydrophobic and non-specific interactions with the gold surface of the sensor-chip. This leads to significant interaction with the sensor followed by strong adhesion and consequent difficulty in removal. To overcome this limitation, a series of functionalization strategies of the gold sensor surface were evaluated to minimize the interaction of the protein of interest with the surface.

The surface-protein interactions, excluding the contribution of thiol groups, are mainly driven by electrostatic forces. Therefore, in considering which strategy is the best to functionalize the gold sensor, it is first of all necessary to evaluate the surface charge of the molecule of interest under the

chosen experimental conditions (i.e., pH value and ionic strength of the medium). Indeed, although in some specific cases finding the right functionalization is very complex, in

general the gold sensor must be functionalized with molecules having the same charge that the protein of interest has at the specific experimental conditions used.

The prime example is given by bovine serum albumin (BSA). At pH = 7, BSA has a mostly negatively charged surface. Hence, the BSA proves to be an excellent choice of functionalization of the gold sensor-chip and therefore allowing to measure the D of proteins with negatively charged surface. On the contrary, for example, positively charged surfaces can be obtained using poly-L-lysine. Again, the reason behind the choice of either BSA or poly-L-lysine to functionalize the sensor is their different charge.

5.1 **4.1 Functionalization workflow**

4.1.1 Activation of the gold surface:

The goal of the activation step is to create a reactive self-assembled monolayer (SAM) on the gold surface. The materials needed are the following:

- Dimethyl sulfoxide (DMSO);
- Di(N-succinimidyl)3,3'-dithiodipropionate (DTPS);
- Small flat bottom container with cap;
- Gold sensor-chip;
- Nitrogen;
- Self-locking tweezers.

The SAM is formed by DTPS, a homobifunctional, amine-reactive cross-linker. The procedure is the following (Fig. S5):

- Transfer a 5 mM DTPS solution in 99% DMSO into the flat bottom container. Pay attention on the height of the solution inside the container, it must be high enough to allow total immersion of the gold
sensor-chip.
- Immerse the clean sensor-chip in the solution using the self-blocking tweezers and place it with the gold layer facing up.
- Create an inert nitrogen atmosphere inside the container to remove the oxygen and leave it in the dark to avoid problems due to oxidation or photoinduced processes, hence maximising the formation of the spontaneous covalent bond between the gold and the DTPS.
- The incubation of the sensor should last for 36 hours or more. The disulphide linkage of DTPS chemisorbs rapidly to gold surfaces and the linkage formed is very stable. The active N-hydroxysuccinimide (NHS) groups on either end of DTPS are reactive towards primary amine groups (e.g. lysine side-chain in proteins).
- After 36 hours (or more), remove the sensor from the container, wash it sequentially with DMSO, MilliQ water and ethanol and dry it with nitrogen gas.
- After these steps insert it into its dedicated slot in the instrument.

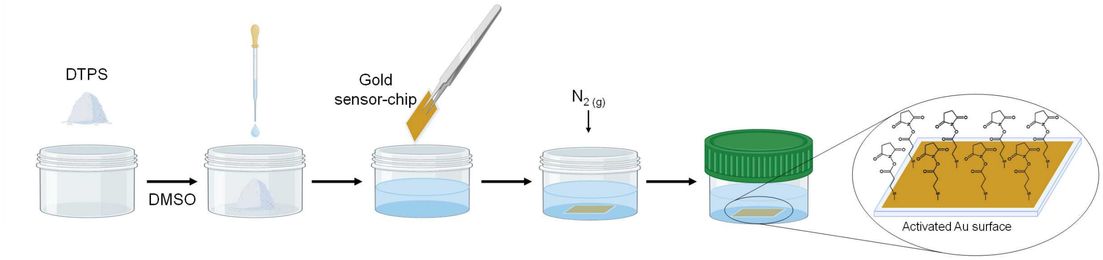

Fig. S5 Scheme of the SPR sensor-chip activation. First, a 5 mM DTPS solution in 99% DMSO is prepared into a flat bottom container. Then, the sensor-chip is put into the solution with the gold side facing up. The container is then filled with an inert N_2 atmosphere to ensure the removal of air on top of the solution. The container is stored at room temperature and in the dark for at least 36 hours.

4.1.2 Immobilization process:

The aim of the immobilization step is to exploit the reactivity of the N-succinimide groups to create a molecular layer on the previously activated surface:

- Prepare a 10 µM solution of the chosen protein (e.g. BSA) in MilliQ for functionalization.
- To increase the yield of the reaction, protein solutions must have a pH equal to the isoelectric point
(IEP) of the protein. For example, the IEP of BSA is at pH 4.5 – 5. Therefore, adjust the pH of the protein solution accordingly.
- Inject the protein solution inside the instrument to let it flow over the activated surface into the flow
cell. Let the injection last for at least 15 minutes at a speed of 20 µL/min. MilliQ water can be used as running buffer.

The primary amines on the surface of the protein will react with the NHS esters on the gold surface leading to stable amide bonds formation. The layer obtained is made of protein molecules randomly oriented on the surface.[2,3] Fig. S6 shows the steps of protein layer formation.

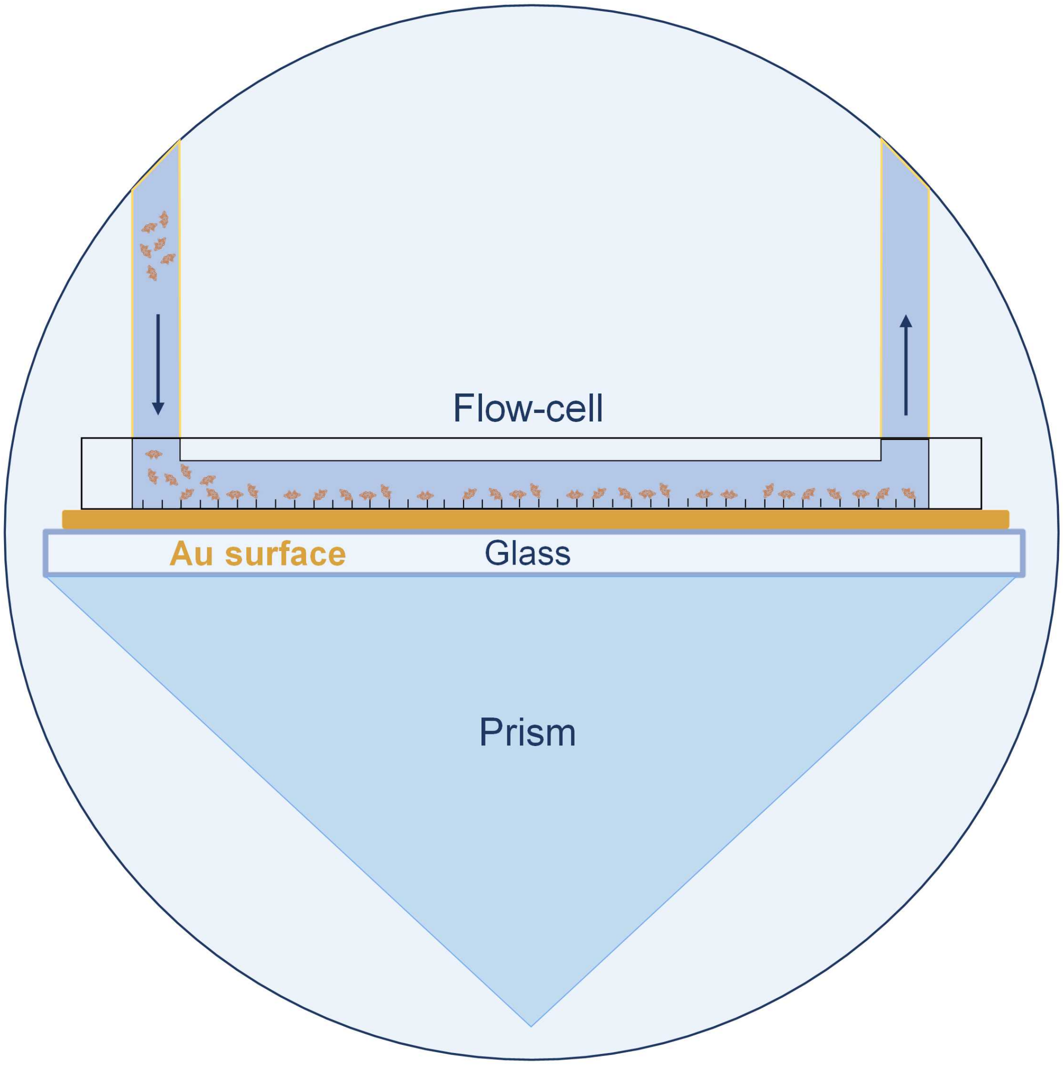

Fig. S6 Schematic representation of the immobilization process occurring inside the flow-cell of the SPR instrumentation. The activated sensor-chip is allocated on the prism into the measurement chamber of the instrument. The solution containing the desired species to be immobilized is injected to flow on the activated gold surface. Its primary amines will react with the DTPS on the surface of the latter and a layer of immobilized molecules will form. Unreacted molecules and byproduct will be then washed with running buffer flowing onto the surface overnight.

4.1.3 Capping/Deactivation:

As there may be some locations where the NHS has not reacted, it is necessary to deactivate the reactive portions of the surface. This step is necessary to prevent the analyte, which will be tested later, to bind covalently to the surface. To deactivate the surface, an injection of a 1 M ethanolamine solution for 15 min at 20 µL/min is normally used.

4.1.4 Equilibration of the sensor-chip surface:

If needed, change the running buffer with the buffer chosen to perform subsequent experiments. To equilibrate the newly obtained surface, let the running buffer flow overnight at a flow rate of 20 µL/min.

6 **5. Running an experiment**

The analyte is injected into the SPR microfluidic system at a flow rate ranging from 5 to 20 µL/min (this may slightly change depending on the tubing i.d. and length available). The running buffer must be the same as the buffer used to dissolve the analyte. Each experiment at each flow rate should be run at least in triplicate in order to obtain a mean and a standard deviation of the D of the molecule of interest. Injection of the analyte into a flow cell having two separate channels counts as a duplicate, so the number of injection to reach at least a triplicate depends on the instrumental setting available.

The injection profile is set to let the analyte flow at the chosen speed for enough time (e.g., 3 minutes or more) to ensure that the SPR signal reaches saturation. Between every experiment, the system is rinsed with the buffer, waiting for the SPR signal to return to the baseline, indicating that the sample has left the flow cell and the system returned to the starting conditions.

Analyte diffusion must occur only during injection into the tubing that connect the loop to the flow cell and not during the loading step. To ensure that no diffusion occurs during the loading step, small air bubbles (1 to 5 µL each) can be used to confine the plug of analyte during its transport inside the loop (Fig. S7). Air bubbles must be discarded before the injection phase, so that they are not included in the section of the microfluidic system leading to the flow cell. Analyte solution consumption is in the order of microliters and it is proportional to the loop size. It is also possible to perform injections in partial loop filling so as to save sample in the case of expensive/precious samples not available in large quantities.

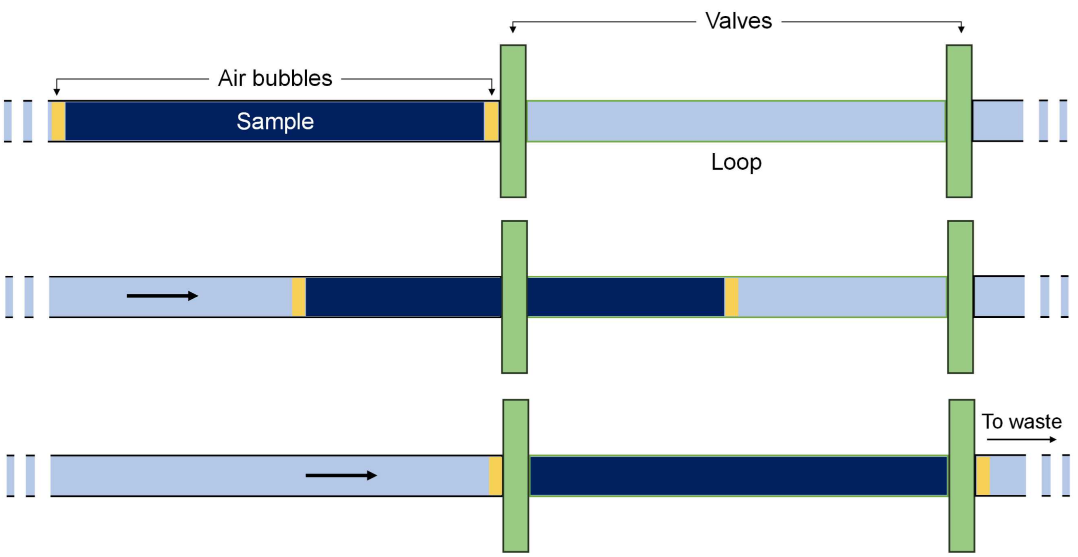

Fig. S7 General sample loading profile used for the SPR experiments. During the loading step, the sample is confined between two low-volume air bubbles to prevent its diffusion before the injection. The air bubbles are discarded before the injection of the sample in the channel leading to the flow-cell where the measurement occur.

Protocol references

[1] G.A. Zingale, I. Pandino, A. Distefano, N. Tuccitto, G. Grasso, A novel SPR based method for measuring diffusion coefficients: From small molecules to supramolecular aggregates, Biosens. Bioelectron. X 13 (2023) 100306. https://doi.org/10.1016/j.biosx.2023.100306.

[2] A. Distefano, G. Antonio Zingale, G. Grasso, An SPR-based method for Hill coefficient measurements: the case of insulin-degrading enzyme, Anal. Bioanal. Chem. (2022). https://doi.org/10.1007/00216-022-04122-3.

[3] G. Grasso, M. Fragai, E. Rizzarelli, G. Spoto, K.J. Yeo, A New Methodology for Monitoring the Activity of cdMMP-12 Anchored and Freeze-dried on Au (111), J. Am. Soc. Mass Spectrom. 18 (2007) 961–969.
