# Supplementary figures and images for: Label-free determination of diffusion coefficients at the nanoscale through modelling of the Surface Plasmon Resonance signal
Source: PLoS One. 2025 Jan 7;20(1):e0312594. doi: 10.1371/journal.pone.0312594 (PMC11706461; doi:10.1371/journal.pone.0312594)

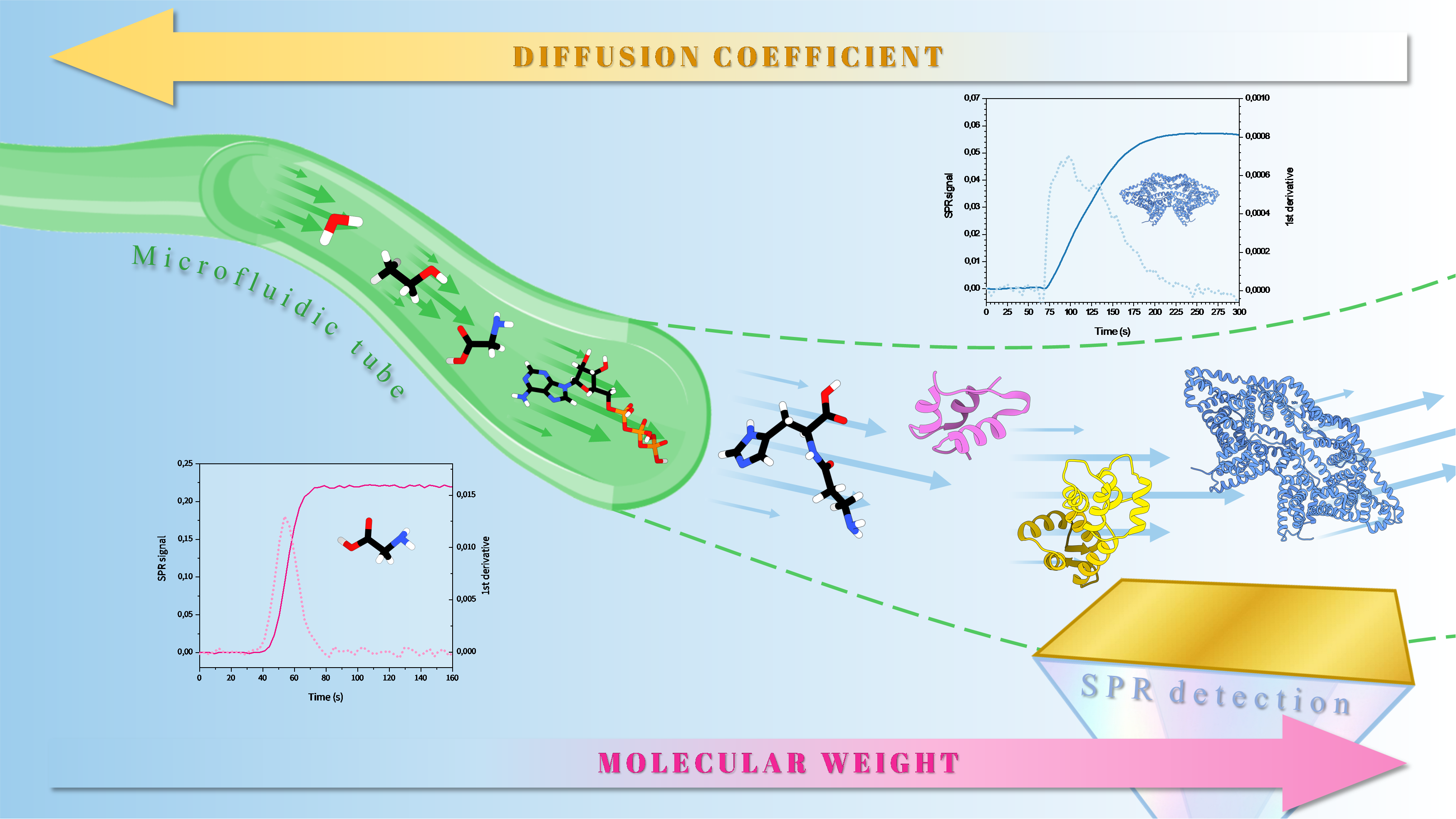

Supplement: S1 Graphical abstract — (TIF) [file pone.0312594.s002.tif]
